# Standardized volumetric 3D-analysis of SPECT/CT imaging in orthopaedics: overcoming the limitations of qualitative 2D analysis

**DOI:** 10.1186/1471-2342-12-5

**Published:** 2012-03-29

**Authors:** Michael T Hirschmann, Christopher R Wagner, Helmut Rasch, Johann Henckel

**Affiliations:** 1 Department of Orthopaedic Surgery and Traumatology, Kantonsspital Bruderholz, CH-4101 Bruderholz, Switzerland; 2 OrthoImagingSolutions Ltd., London, UK; 3 Institute for Radiology and Nuclear Medicine, Kantonsspital Bruderholz, CH-4101 Bruderholz, Switzerland; 4 Department of Musculoskeletal Surgery, Imperial College, London, UK

## Abstract

**Background:**

SPECT/CT combines high resolution anatomical 3D computerized tomography (CT) and single photon emission computerized tomography (SPECT) as functional imaging, which provides 3D information about biological processes into a single imaging modality. The clinical utility of SPECT/CT imaging has been recognized in a variety of medical fields and most recently in orthopaedics; however, clinical adoption has been limited due to shortcomings of analytical tools available. Specifically, SPECT analyses are mainly qualitative due to variation in overall metabolic uptake among patients. Furthermore, most analyses are done in 2D, although rich 3D data are available. Consequently, it is difficult to quantitatively compare the position, size, and intensity of SPECT uptake regions among patients, and therefore difficult to draw meaningful clinical conclusions.

**Methods:**

We propose a method for normalizing orthopaedic SPECT/CT data that enables standardised 3D volumetric quantitative measurements and comparison among patients. Our method is based on 3D localisation using clinically relevant anatomical landmarks and frames of reference, along with intensity value normalisation using clinically relevant reference regions. Using the normalised data, we describe a thresholding technique to distinguish clinically relevant hot spots from background activity.

**Results:**

Using an exemplar comparison of two patients, we demonstrate how the normalised, 3D-rendered data can provide a richer source of clinical information and allow quantitative comparison of SPECT/CT measurements across patients. Specifically, we demonstrate how non-normalized SPECT/CT analysis can lead to different clinical conclusions than the normalized SPECT/CT analysis, and that normalized quantitative analysis can be a more accurate indicator of pathology.

**Conclusions:**

Conventional orthopaedic frames of reference, 3D volumetric data analysis and thresholding are used to distinguish clinically relevant hot spots from background activity. Our goal is to facilitate a standardised approach to quantitative data collection and comparison of clinical studies using SPECT/CT, enabling more widespread clinical use of this powerful imaging tool.

## Background

SPECT/CT combines a high resolution anatomical 3D computerized tomography (CT) and a single photon emission computerized tomography (SPECT) as functional imaging, which provides 3D information about biological processes into a single imaging modality. Along with the fusion of these images the metabolic activity in a region of interest can be accurately allocated to specific anatomical areas [1-3]. Until recently, registration of the functional images (SPECT) with the structural images (CT) has been difficult due to SPECT's poor spatial resolution (3-10 mm) and its high variability in identifying anatomical landmarks [4,5]. With the advent of integrated SPECT/CT technology, where the SPECT data is obtained in the same coordinate frame as the CT, image fusion has significantly improved [6].

Although SPECT/CT has proven useful as a diagnostic tool in a variety of medical fields and most recently in orthopaedics [1,7-11], its use has a number of limitations. Firstly, SPECT tracer uptake values are only valid for the individual patient investigated and cannot easily be compared between patients. Therefore, it is difficult to draw clinically useful conclusions on intensity and size of SPECT regions. Several authors have introduced methods of quantification of SPECT data to minimize subjective tracer interpretation, however to date no quantitative analysis has been described in orthopaedic patients [12-16]. Secondly, although 3D-data are generally obtained, analysis is routinely performed only in 2D (ROI analysis) [11]. Thirdly, accurate and reproducible localization of increased or decreased tracer uptake in 3D is difficult to achieve [10,11]. Fourthly, to date clinical correlation of the biology, the biomechanics and the function of joint pathology has not been sufficiently studied in orthopaedics.

### Enhanced tracer uptake regions

The pattern and intensity (increased, decreased) of tracer distribution in a SPECT scan gives information on bone and soft tissue pathologies. Radionuclide tracers are chosen that preferentially bind to a specific metabolic activity (e.g., technetium-labeled diphosphonate for osseous activity), and injected intravenously 3-4 hours before the SPECT and CT imaging. Regions of increased tracer uptake (which we term a "hotspot") may signal a metabolically active region and provide a diagnostic aid. Current analysis, however, is limited to this relative comparison (increased or decreased) as opposed to quantifying the intensity of the tracer uptake region in a clinically significant way.

A key hurdle is the significant inter- and intra-patient variation in the metabolic uptake of radionuclide tracers. Variation of more than 10 times in the average SPECT intensity is not uncommon between patients. Because of this variation, it is difficult to identify a clinically relevant SPECT intensity threshold to distinguish regions of interests from normal background variation.

## Methods

The purpose of this paper is to propose a method to normalize the 1) location, 2) size, and 3) intensity of altered tracer uptake regions to allow quantitative comparison of orthopaedic SPECT/CT scans. Our method uses standardized orthopaedic frames of reference for orientation, and 3D volumetric data interpretation and thresholding to distinguish clinically relevant hot spots from background activity. Along with its introduction we strive to facilitate data collection and comparison of clinical studies using SPECT/CT. In this section we present the normalization method details. We then provide an exemplar patient comparison as a demonstration of the method's use in the Results.

### Data analysis

The proposed normalization techniques and analysis can be carried out with any software that can reorient 3D data and quantify intensities of the SPECT voxels. We have implemented the software tools necessary for the exemplar patient comparison using a collection of open-source tools. The software was written in Python (v2.6, http://python.org/), using the Grassroots DICOM library (GDCM v2.0.12, http://gdcm.sourceforge.net/) to read the SPECT and CT DICOM files exported by the SPECT/CT machine, the Visualisation Toolkit (VTK v5.4.2, Kitware, http://vtk.org/) library for surface and volume rendering, and the NumPy (http://numpy.scipy.org/) Python library for data analysis. Note that a slight modification was required to the GDCM library to overcome an idiosyncrasy in the SPECT DICOM data for the specific SPECT/CT machine used (Symbia T16, Siemens).

### Normalisation of position: orthopaedic reference frames

To reliably locate and describe the position of a region of altered metabolic activity it is essential that the reference system is well defined. Further, to overcome the relative position and orientation of the patient to the coordinate system of the scanner, the reference system should be based on anatomic landmarks that are identifiable in subsequent scans. The unique combination of anatomic and metabolic information available with SPECT/CT imaging allows the low resolution metabolic information from SPECT to be accurately localised using the high resolution anatomic information from CT.

We use well-recognised standard anatomic landmarks and corresponding frames of reference that relate to the biomechanics of the joint. The landmarks can be manually identified on a combination of CT slices and on the surface of a 3D bone reconstruction from the CT data. These landmarks define a coordinate system specific to the lower limb, independent of scanner-specific coordinates. We can then use this normalised coordinate system to localize the biomechanical and diagnostic SPECT regions of interest. Further, with this normalised coordinate system we can provide normalised views for the 3D bone reconstruction and 2D slices (i.e., "true" antero-posterior views and slices that will be the same perspective relative to the anatomy independent of the patient position and orientation in the scanner).

**Figure 1:**
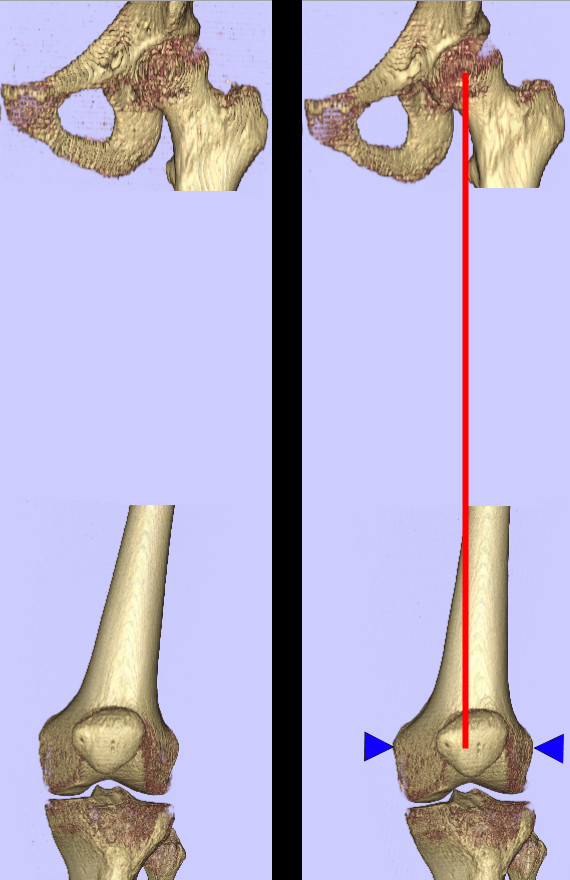
**Femoral anatomic reference frame**. The femur is aligned to an anatomic reference frame, independent of patient position in the scanner, using the mechanical axis determined by the femoral head centre and the centre of the knee defined by the medial and lateral femoral epicondyles. This reference frame provides slice alignment and 3D reconstruction views that are the same for multiple scans of the same patient, as well as comparable views for different patients.

The femoral frame of reference is defined as containing the mechanical axis of the femur and the axis of rotation of the knee, with the origin at the centre of the joint (Figure 1). This frame of reference gives the anteroposterior axis and the mediolateral axis of the femur [17]. The midpoint of a straight line joining the surface locations of the epicondyles is taken as the centre of the knee. We use the femoral head centre as the proximal landmark defining the mechanical orientation of the femur. This point is defined as the intersection of the diameters of the head in all three planes. The mechanical axis of the femur is then defined as the line passing from the centre of the femoral head to the centre of the knee. We establish an orthogonal reference frame using the cross product of the transepicondylar line and the mechanical axis to define the anteroposterior axis, and the cross product of the anteroposterior axis and the mechanical axis to define the mediolateral axis.

**Figure 2:**
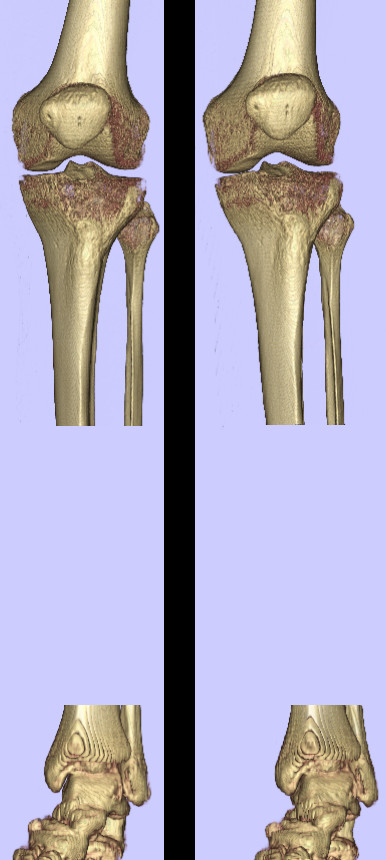
**Tibial anatomic reference frame**. The tibia is aligned similar to the femur, treating the knee joint as a simple hinge. The centre of the talus is used as a distal landmark.

The tibial orthogonal reference frame is constructed to treat the knee as a simple hinge, so that tibial sagittal planes are the same as the femoral sagittal planes (Figure 2). Specifically, the mediolateral axis is taken to be the same as the femoral medioateral axis, the anteroposterior axis is defined as the cross product of the mediolateral axis and the line between the knee centre (as defined above) proximally and the centre of the talus distally, and the mechanical axis as the cross product between mediolateral axis and the anteroposterior axis.

### Normalisation of size and intensity: clinically neutral reference region

Here we propose a method for normalising the SPECT intensities between patients to allow for quantitative analysis, potentially increasing the value of SPECT imaging. Direct comparison of raw SPECT intensities is known to be problematic, due to significant variation among patients in overall uptake levels. Normalisation techniques that rely purely on the distribution of SPECT intensities, such as scaling by the maximum value, or normalising using the mean and standard deviation, are also problematic. These techniques can be biased by the fraction of tissue related voxels as compared to free air voxels (affected by patient size and field of view), the presence of an intense hotspot, or an overall inflammatory uptake response.

What we propose is to use the anatomic information provided by the CT component to establish, prior to analysis, a clinically relevant neutral region. For example, when analysing intensities relating to knee joint pain, we can define a volume in the middle of the femoral shaft (which is unrelated to the articular surface) as a reference region. The distribution of SPECT intensities from this region can be then be used to normalize other values. This approach avoids the biases associated with patient size, field of view, and normalising solely on the global distribution of SPECT intensities. It also takes advantage of the unique combination of anatomic and metabolic information offered by SPECT/CT imaging.

The optimal method to normalize hotspot intensities using reference region intensities, resulting in an analysis with the best correlation with clinical outcomes, remains an open research question and likely depends on the specific analysis conducted. Potential approaches include scaling intensities to the mean of the reference region, offsetting intensities with the mean of the reference region, using the standard deviation of the values in the reference region as a scaling parameter, or some combination of these techniques. Hotspots can then be compared in a statistical manner, using a *t*-test to compare the distribution of values in a hotspot with the values in the reference region. Intensity thresholds can also be defined in a straightforward way, such as more than *n *standard deviations away from the reference region mean.

### Thresholding for volumetric 3D tracer uptake analysis and visualisation

**Figure 3:**
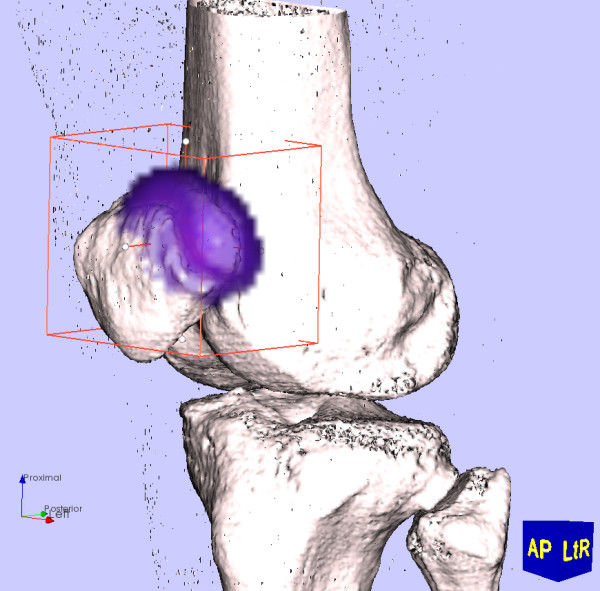
**Thresholding for analysing SPECT data**. Using a threshold cutoff combined with an easy-to-use rectangular region selector allows for straightforward identification of hotspot voxels for further analysis.

Having established clinically relevant intensity thresholds using the method described above, we can use thresholding (an algorithmically simple operation) to distinguish altered tracer uptake voxels from background voxels. This avoids the difficult segmentation procedure normally associated with quantitative analysis of medical images. The threshold also enables simple volumetric analysis within a region. For example, one can use a rectangular volume (which is straightforward to define) to define an initial region, then use the threshold to distinguish altered tracer uptake voxels from background voxels within the region (Figure 3).

**Figure 4:**
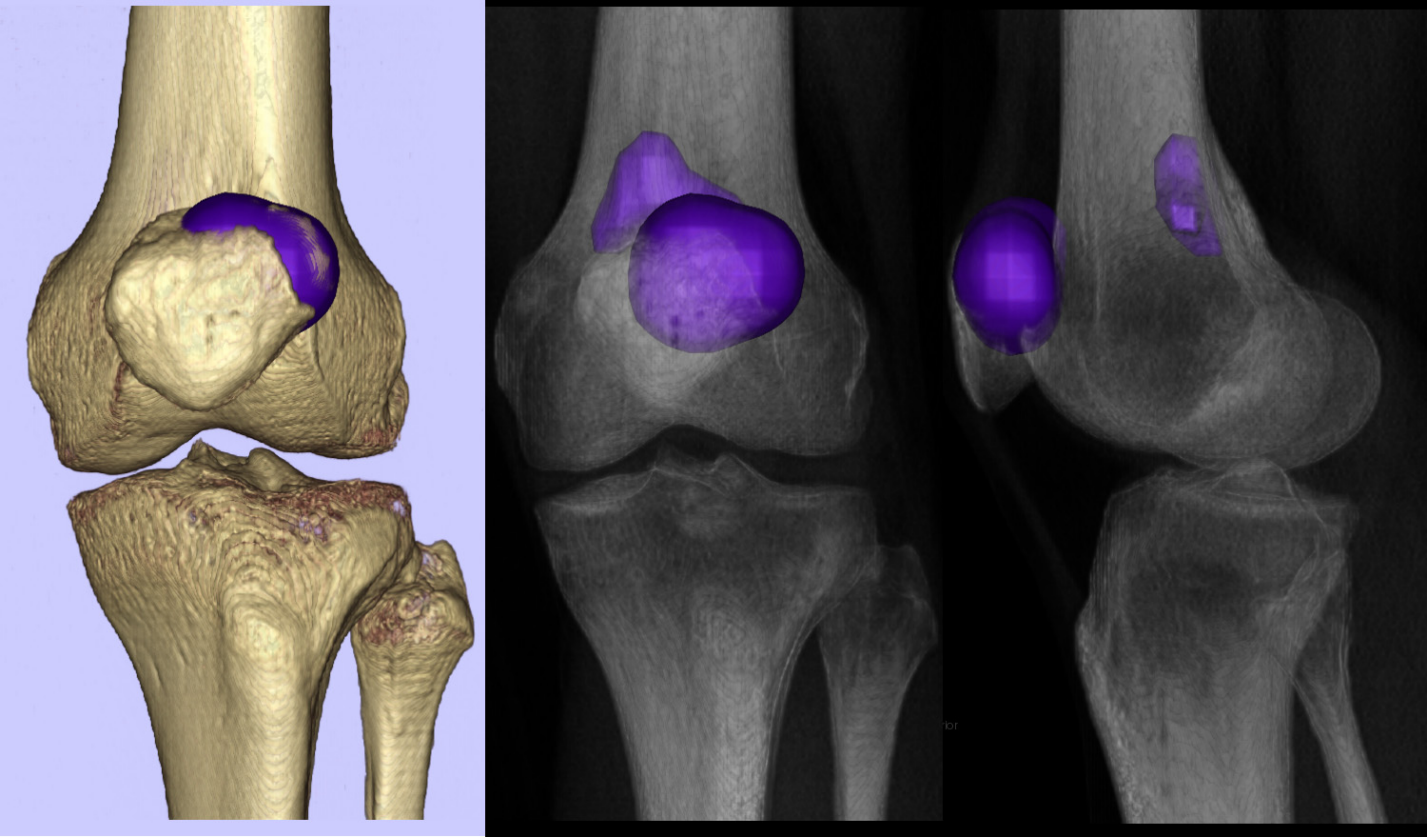
**Rendering SPECT region of interest**. Once a clinically relevant SPECT intensity threshold has been established, we can render a hotspot surface at that threshold for a visual appreciation of the hotspot size and position.

This clinically relevant intensity also defines a boundary for the 3D surface reconstruction (i.e., isosurface rendering) of the SPECT region of interest (Figure 4). The SPECT region boundary surface can be rendered with the 3D bone surface reconstruction, enabling views from any perspective including the normalized views described above. This can aid the clinician's understanding of the position of the uptake region, which is of particular use when informing a surgical procedure.

## Results

Here we present an exemplar analysis of SPECT/CT scans of two patients presented to our hospital, demonstrating the establishment of orthopaedic frames of reference, the localisation of SPECT tracer uptake regions, the normalisation of the intensities (based on the intensities in a neutral region in the femoral shaft) and a quantitative comparison of the size and intensity of the SPECT voxels in patients with symptomatic patellofemoral joints.

### Patient description

Both patients presented to our hospital complaining about knee pain with the history and clinical examination suggestive of patellofemoral disease. We decided to demonstrate the proposed method on these two patients because most imaging modalities are limited when it comes to the patellofemoral joint. Both patients have been diagnosed with patellofemoral osteoarthritis, but show a different distribution of osteoarthritis. In one the cause is considerably a slight trochlear dysplasia and the other one is idiopathic.

### SPECT/CT imaging

For both patients, Tc-99m-HDP-SPECT/CT was performed using a hybrid system (Symbia T16, Siemens, Erlangen, Germany) that consists of a pair of low energy, high-resolution collimators and a dual-head gamma camera and an integrated 16x0.75-mm slice-thickness CT. A commercial 700 MBq Tc-99m HDP tracer (CIS Bio International Sur Yvette, France) was used. SPECT imaging was performed at 3-4 hours post injection, followed by a CT without moving the patient. In addition, 2D perfusion images were obtained in the initial 60 seconds post injection followed by 2D blood pool images 2-5 minutes later.

### Non-normalized analysis and visualisation

**Figure 5:**
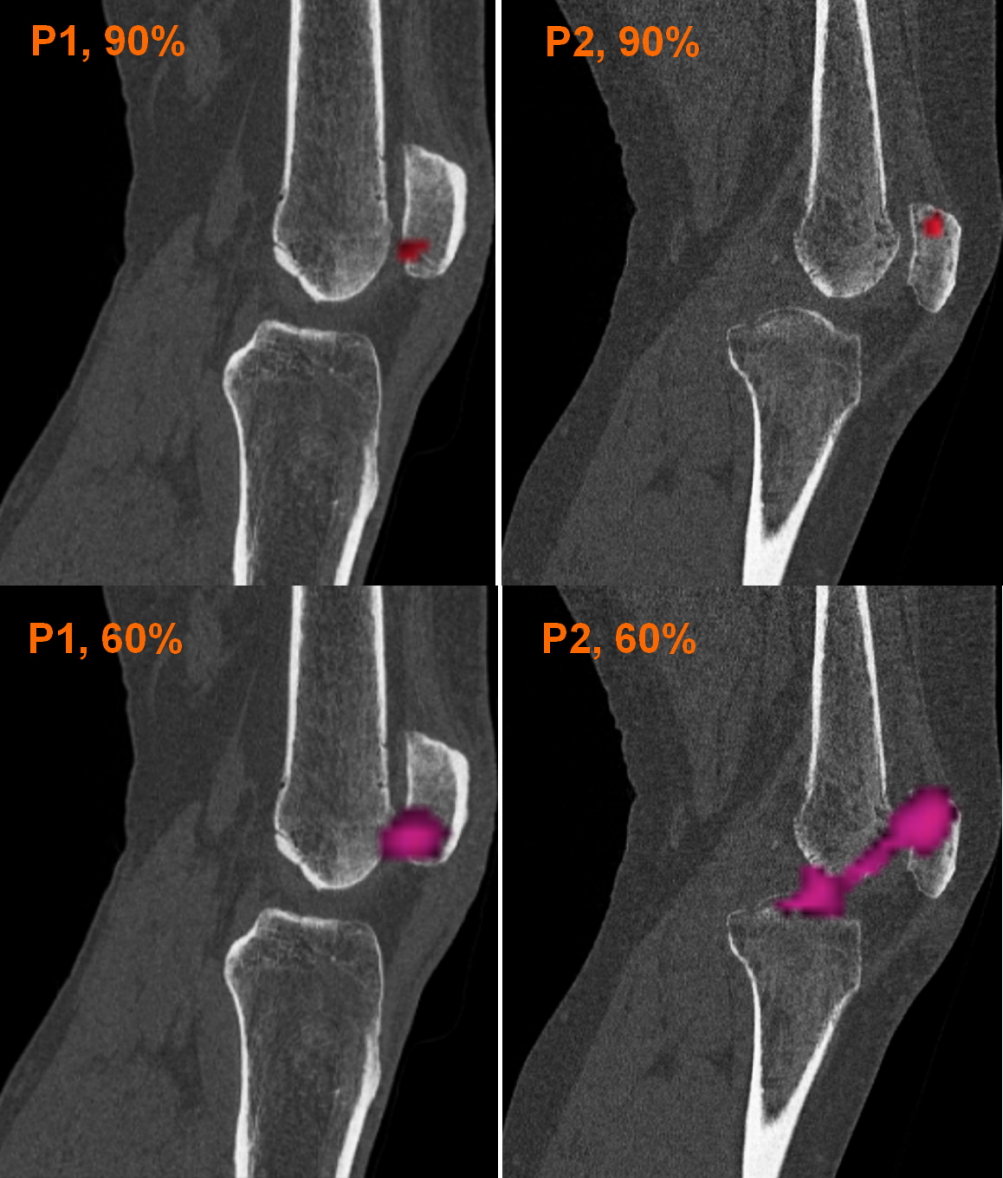
**Non-normalized SPECT renderings**. Renderings of the SPECT uptake region for patient 1 (left column) and patient 2 (right column) at 90% maximum SPECT intensity (top row) and 60% maximum SPECT intensity (bottom row). Without a method to identify a clinically relevant threshold intensity, drawing clinical conclusions is problematic.

Initial SPECT renderings (threshold SPECT voxels at 90% of maximum intensity) show similar tracer uptake patterns, in particular the size and intensity of the altered tracer uptake region (Figure 5), indicating isolated patellofemoral disease in both patients. However, thresholding at a lower value (60% of maximum) reveals a different uptake extent. At this lower threshold, P2's uptake region extents into the tibiofemoral joint, while P1's hotspot is localized within the patellofemoral joint only. Without a normalization procedure to inform a clinically relevant threshold, it is difficult to determine whether P2's observed uptake is part of normal physiological variation in tracer uptake or is an indication of pathology.

### Normalisation of position: orthopaedic reference frames

**Figure 6:**
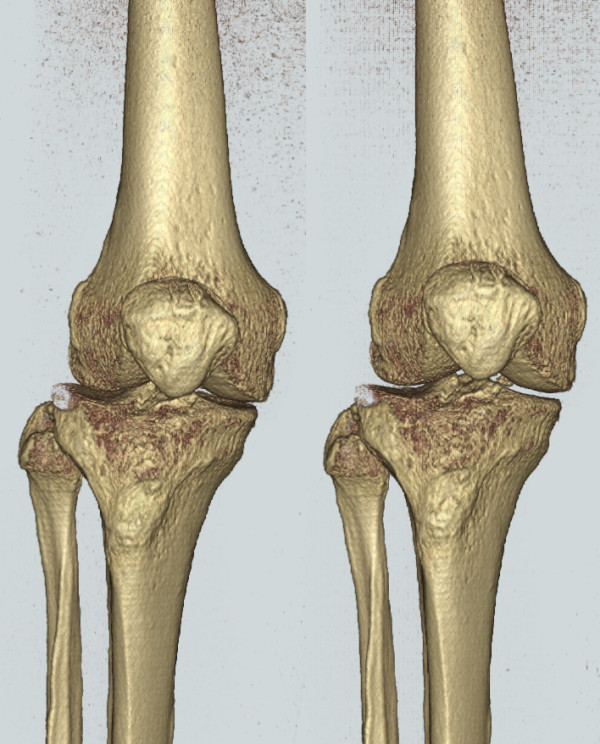
**Patient 2 femur before and after realignment**. Before (left) and after (right) realignment of patient 2 femur to anatomic based reference frame. Note change of more than 5 degrees in axial alignment.

The femur and tibia of both SPECT/CT datasets were reoriented into the normalised (anatomic-based) reference frames described above. Note that for P2, this resulted in a realignment of the femur of more than 5 degrees, an important correction if the scanner-based reference frame was used for orthopaedic measurements (Figure 6). All following figures of slices and reconstructions are presented in the normalised perspectives (e.g., "true" AP and lateral views).

### Normalisation of size and intensity: clinically neutral reference region

**Figure 7:**
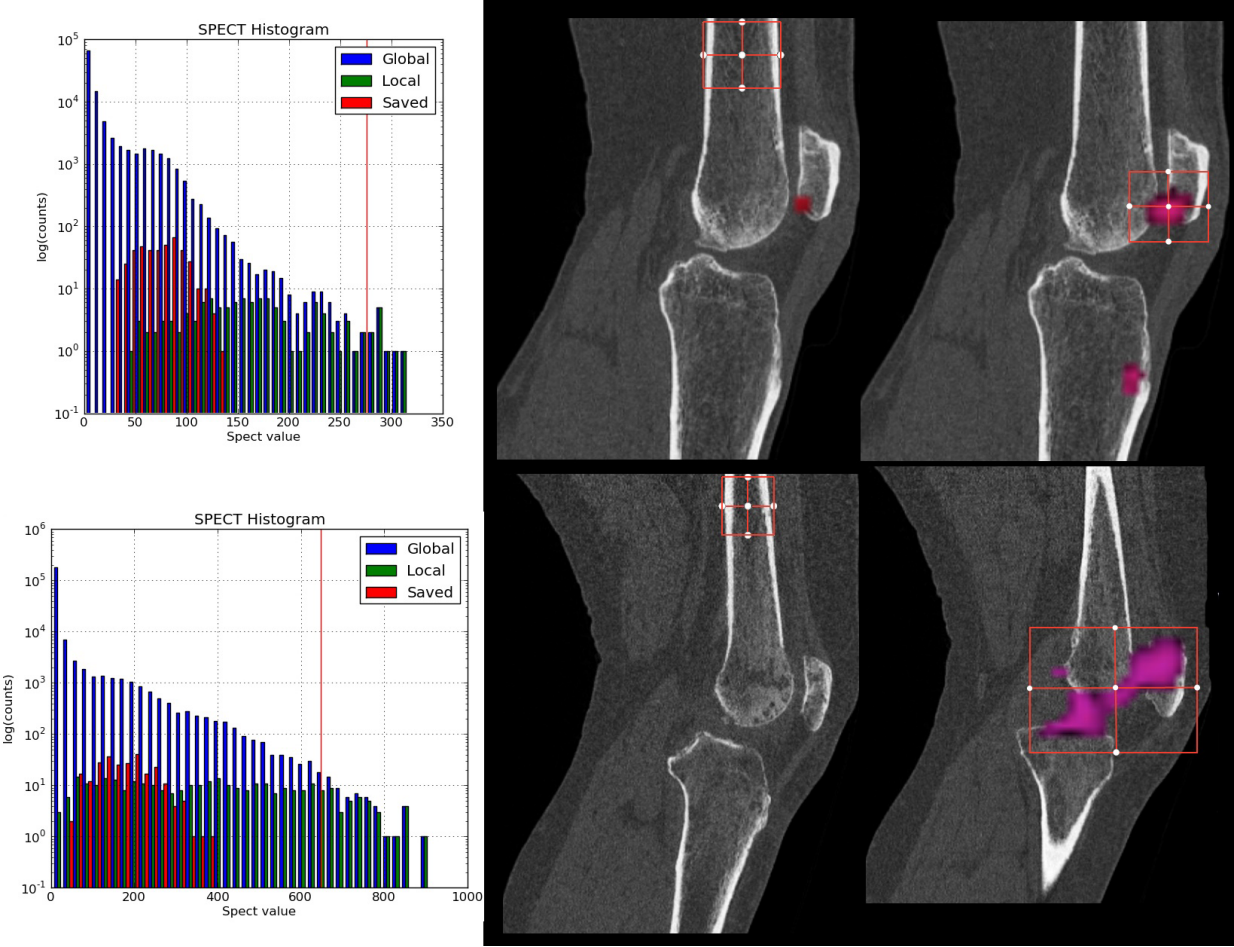
**SPECT intensities in reference region and region of interest**. Comparison of the tracer uptake intensities in the region of interest (right column) relative to the intensities in a reference region (middle column) for patient 1 (top row) and patient 2 (bottom row). The SPECT histograms (left column) illustrate SPECT tracer distribution of the reference region ("saved") in comparison to the SPECT region of interest ("local") and the whole dataset ("global").

We used a region in the middle of the femur to establish a clinically neutral reference region (Figure 7). The SPECT intensities within this neutral region were then used as a baseline distribution from which we could compare the SPECT intensities on the articular surfaces. For this comparison, we used three times the average intensity in the reference region to signify a clinically relevant level of increased tracer activity. (Again, the clinically optimal relationship between the reference region, the hotspot and clinical outcomes remains an area of active research.)

### Clinical implications

**Figure 8:**
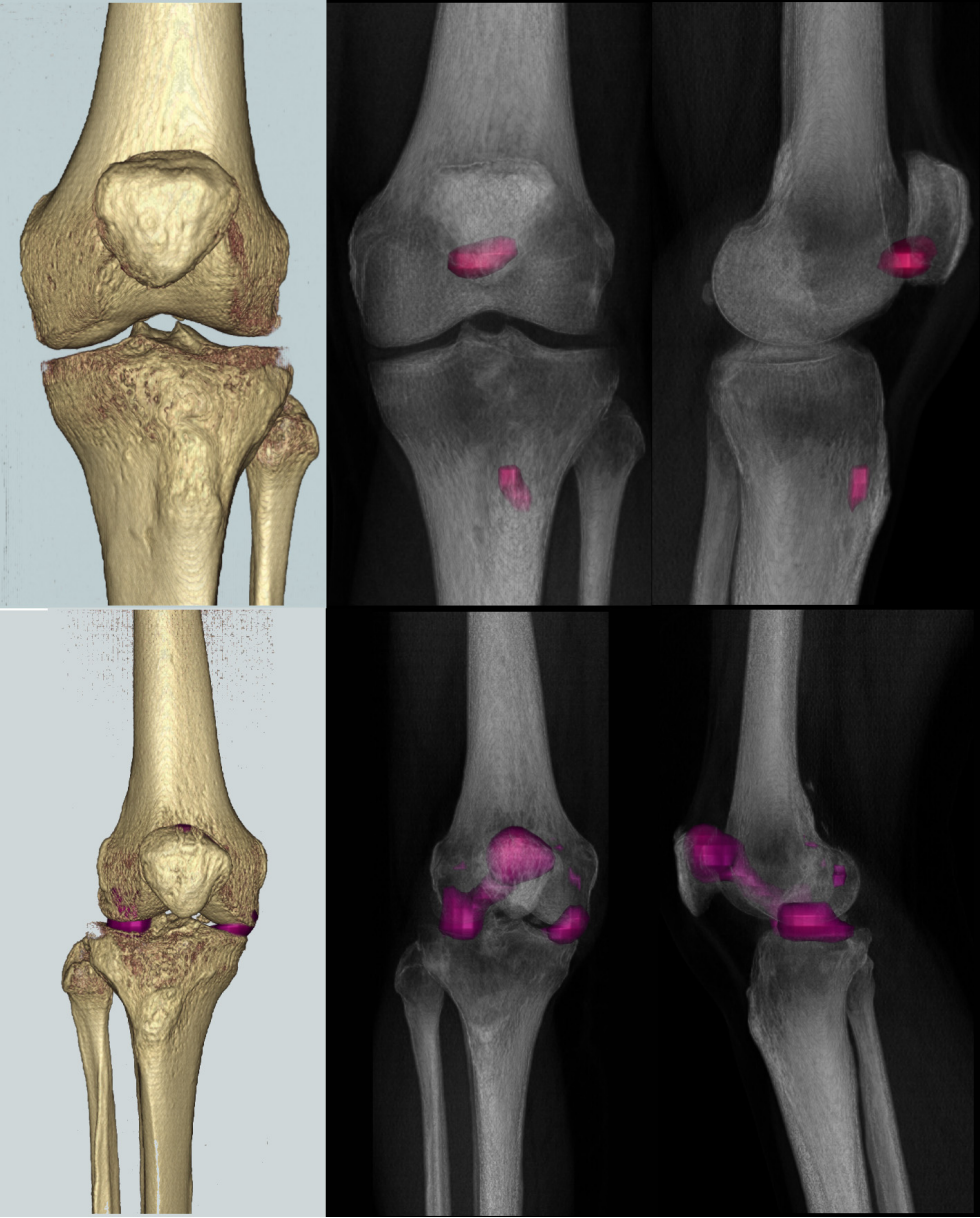
**3D reconstructions of SPECT and bone surface**. The SPECT hot spots are demonstrated at our chosen SPECT threshold (three times mean of the reference region) as a 3D surface reconstruction (left) and radiolucent (right) views (patient 1 top, patient 2 bottom row).

**Figure 9:**
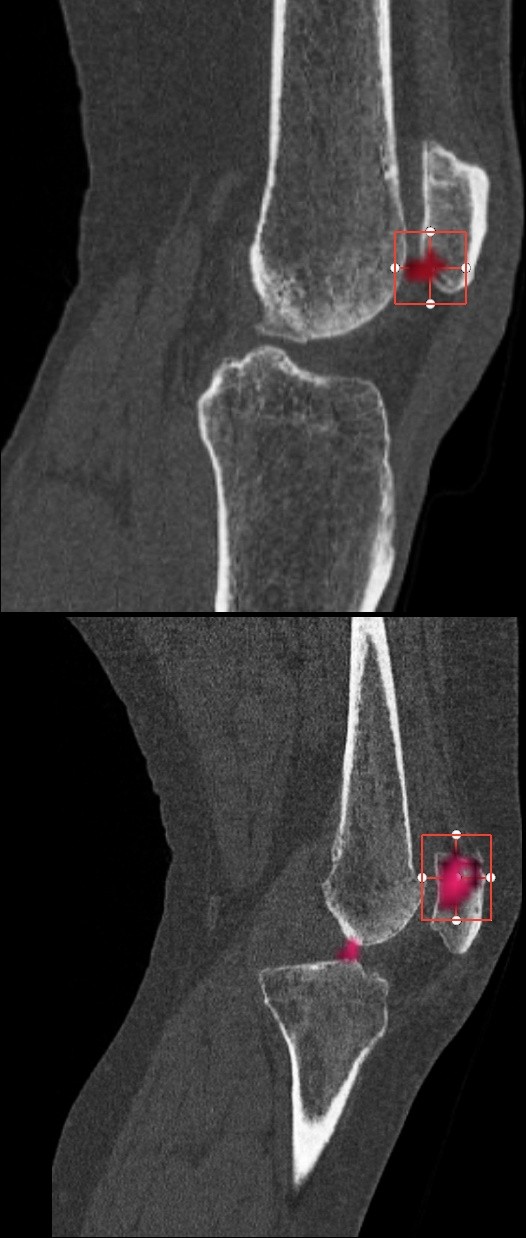
**SPECT uptake region analysis**. Using a higher threshold (four times the mean of the reference region) showed a higher value for patient 2's uptake region (bottom figure, upper patellar pole) as compared to patient 1, (top figure, lower patellar pole).

Using the above determined normalized threshold, we were able to analyze and re-render the SPECT data at what we considered a clinically significant threshold (Figure 8). This rendering supports the clinical diagnosis of isolated patellofemoral disease in P1 and bi-compartmental disease in P2. Comparison of the intensities of the maximum value in the region of interest against the intensity of the reference region further supported the conclusion of P2's increased degeneration. P1's maximum was less intense (4.55 times the mean reference intensity) than P2's (5.56 times the mean reference intensity). Similarly, comparing the mean value of the region of interest (using four times the mean of the reference region to focus on the patellofemoral hotspot in each patient) revealed that P1's mean was less intense (P1: 4.19 vs. P2: 4.49) (Figure 9). Finally, comparing the number of SPECT voxels above the clinical threshold show that P2's uptake region is larger in size (volume) than P1's.

## Discussion

We have proposed a novel method of standardized quantitative volumetric 3D-analysis of SPECT/CT imaging to overcome the limitations of qualitative 2D analysis. A standardised approach to quantitative volumetric 3D data collection using SPECT/CT enabling analysis of the size, the intensity and the localisation of enhanced tracer uptake regions of SPECT/CT, that we put forward as "hotspot", was introduced. The major contributions of our method are the following:

1. Standardised 3D-CT reconstructions using relevant surgical anatomical landmarks are used to define orthopaedic frames of reference for biomechanical orientation [18]. Only after orientation of the data in relation to the mechanical axis of the lower limb can reliable and meaningful information be drawn for the diagnosis and treatment of patients with musculoskeletal disorders [11,19-21]. To our knowledge the proposed method offers for the first time the chance to relate the information of joint mechanics (CT) with clinical hotspots as biologic/metabolic data (SPECT).

Current imaging and image processing techniques are not sufficiently advanced to investigate certain pathologies and compartments of joints. The patellofemoral disease is one such condition, which is difficult to differentiate from the other two compartments of the knee. In addition, it is a typical example for the symbiosis of joint mechanics and biology. This is also relevant for patients following joint replacement surgery [11,18,20,21]. This 3D localisation method enables the surgeon to more clearly identify the clinically relevant hotspots and aid in understanding of the origin of the patient's symptoms.

2. It is widely known that individual SPECT hotspots vary greatly among patients. The normalizing techniques described here allow comparison of quantitative measurements amongst different patients. It may contribute to the understanding of pathologies and clinical value in clinical trials using SPECT/CT [11,20,22,23]. Using our proposed method of image analysis and processing we hope to distinguish clinically relevant hotspots from normal background activity. Normalization approaches are biased by the patient size, the field of view and metabolic activity in areas outside the region of clinical interest [11]. The establishment of clinically neutral reference regions prior to analysis addresses these problems. The limitations of the conventional approach to SPECT data normalisation, which relies on the distribution of SPECT intensities, scaling by the maximum values or normalisation using the mean or standard deviation are also avoided. However, the optimal method to normalize hotspot intensities using reference region intensities is an open research question. It may depend on different methods of data analysis [11,20,22,23]. The optimal approach to scaling of intensities has to be investigated in clinical studies. We suggest studies looking into the normalisation of hotspots and offsetting intensities with the mean of the reference region. Using the standard deviation of the values in the reference region as a scaling parameter or some combination of these techniques might also be helpful to improve diagnostics further [21]. Hotspots can then be compared in a statistical manner to compare the distribution of values within a hotspot with that of the reference region. Intensity thresholds can also be defined as a factor of the standard deviation from the reference region mean.

This proposed method of quantitative 3D analysis is novel and contrasts previous clinical studies using SPECT or SPECT/CT in orthopaedics, where the SPECT intensity and size of hotspots was graded using a Likert scale (0-10) or descriptively analysed [11,20-23]. To date, most authors consider the area of maximum tracer activity as the area of interest analysing the peak values only [11,21]. However, this method neglects the lower intensity SPECT values and may lead to a decrease in sensitivity and specificity in specific clinical conditions. It may also be case that the pattern of uptake provides the surgeon with more clinically relevant information leading to a more accurate diagnosis.

## Conclusions

We propose a standardized method of analysing the location, size and the intensity of SPECT/CT tracer uptake regions ("hotspots"). Conventional orthopaedic frames of reference, 3D volumetric data analysis and thresholding are used distinguish clinically relevant hot spots from background activity. We demonstrated how the normalised, 3D-rendered data may provide a richer source of clinical information and allow quantitative comparison of SPECT/CT measurements across patients. This article gives a detailed description on the proposed method, but its clinical utility will be investigated in further clinical studies.

## Competing interests

The authors declare that they have no competing interests.

## Authors' contributions

MTH, CRW, HR and JH participated in the design of the study, performed the data analysis and drafted the manuscript. All authors read and approved the final manuscript.

## Pre-publication history

The pre-publication history for this paper can be accessed here:

http://www.biomedcentral.com/1471-2342/12/5/prepub
